# Microbiology of healthcare-associated infections and the definition accuracy to predict infection by potentially drug resistant pathogens: a systematic review

**DOI:** 10.1186/s12879-015-1304-2

**Published:** 2015-12-11

**Authors:** Teresa Cardoso, Mónica Almeida, Jordi Carratalà, Irene Aragão, Altamiro Costa-Pereira, António E. Sarmento, Luís Azevedo

**Affiliations:** Intensive Care Unit (UCIP), Hospital de Santo António, Oporto Hospital Center, University of Oporto, Largo Prof. Abel Salazar, 4099-001 Porto, Portugal; Neurocritical Care Unit, Hospital de Braga, Sete Fontes, São Vitor, 4710-243 Braga, Portugal; Infectious Disease Service, Hospital Universitari de Bellvitge-IDIBELL, University of Barcelona, Feixa Llarga s/n, 08907 L’Hospitalet de Llobregat, Barcelona, Spain; Department of Health Information and Decision Sciences, Center for Research in Health Technologies and Information Systems (CINTESIS), Faculty of Medicine, University of Porto, Alameda Prof. Hernâni Monteiro, 4200-319 Porto, Portugal; Department of Infectious Diseases, Hospital de São João, University of Porto, Alameda Prof. Hernâni Monteiro, 4200-319 Porto, Portugal

**Keywords:** Healthcare-associated infection, Pneumonia, Bacteraemia, Urinary tract infection, Endocarditis, Intra-abdominal infections, Multidrug resistant pathogens

## Abstract

**Background:**

Healthcare-associated infections (HCAI) represent up to 50 % of all infections among patients admitted from the community. The current review intends to provide a systematic review on the microbiological profile involved in HCAI, to compare it with community-acquired (CAI) and hospital-acquired infections (HAI) and to evaluate the definition accuracy to predict infection by potentially drug resistant pathogens.

**Methods:**

We search for HCAI in MEDLINE, SCOPUS and ISI Web of Knowledge with no limitations in regards to publication language, date of publication, study design or study quality. Only studies using the definition by Friedman et al. were included. This review was registered at PROSPERO Systematic Review Registration with the Number CRD42014013648.

**Results:**

A total of 21 eligible studies with 12,096 infected patients were reviewed; of these 3497 had HCAI, 2723 were microbiologically documented. Twelve studies were on pneumonia involving 1051 patients with microbiological documented HCAI, the application of the current guidelines for this group of patients would result in an appropriate antibiotic therapy in 95 % of cases at the expense of overtreatment in 73 %; the application of community-acquired pneumonia guidelines would be adequate in only 73–76 % of the cases; an alternative regimen with piperacillin-tazobactam or aztreonam plus azithromycin would increase antibiotic adequacy rate to 90 %. Few studies were found on additional focus of infection: endocarditis, urinary, intra-abdominal and bloodstream infections. All studies included in this review showed an association of the HCAI definition with infection by PDR pathogens when compared to CAI [odds ratio (OR) 4.05, 95 % confidence interval (95 % CI) 2.60–6.31)]. The sensitivity of HCAI to predict infection by a PDR pathogen was 0.69 (0.65–0.72), specificity was 0.67 (0.66–0.68), positive likelihood ratio was 1.9 and the area under the summary ROC curve was 0.71.

**Conclusions:**

This systematic review provides evidence that HCAI represents a separate group of infections in terms of the microbiology profile, including a significant association with infection by PDR pathogens, for the main focus of infection. The results provided can help clinician in the selection of empiric antibiotic therapy and international societies in the development of specific treatment recommendations.

**Electronic supplementary material:**

The online version of this article (doi:10.1186/s12879-015-1304-2) contains supplementary material, which is available to authorized users.

## Background

The concept of healthcare-associated infections (HCAI) was proposed in 2002 as a new category to fill the gap between community (CAI) and hospital-acquired infections (HAI) [1, 2]. Since then a broad range of definitions have been used with the definition originally proposed by Friedman et al. [1] being the most commonly used in clinical studies on HCAI [3] - an infection present at hospital admission or within 48 h of admission in patients that fulfilled any of the following criteria:

* received intravenous therapy at home, wound care or specialized nursing care through a healthcare agency, family or friends; or had self-administered intravenous medical therapy in the 30 days before the infection. Patients whose only home therapy was oxygen use were excluded;
* attended a hospital or hemodialysis clinic or received intravenous chemotherapy in the previous 30 days;
* were hospitalized in an acute care hospital for two or more days in the previous 90 days,
* resided in a nursing home or long-term care facility.

Patients with HCAI can represent up to 50 % of all infected patients admitted from the community setting [1, 4–10] and are clearly a growing class due to the increasing age of patients, as this means they have more chronic diseases, require more medical and surgical interventions, are consequently more frequently hospitalized and institutionalized and are thus more at risk of colonization of and infection by multidrug resistant pathogens [11]. Nearly half of all HCAI patients receive antibiotic therapy according to international guidelines for community-acquired infections, with associated high rates of inadequate antibiotic therapy among them [12].

In 2005, the ATS/IDSA recommended a treatment for healthcare associated pneumonia (HCAP) similar to hospital-acquired pneumonia [13], but accumulating evidence suggests that such a broad spectrum antibiotic therapy approach might not be necessary and probably should be avoided in order to prevent the inherent development of resistances [14].

The absence of a unique and consensual definition has led to contradictory results and increasing controversy around the utility of the HCAI concept [15, 16]; the inclusion of different populations lead to different results in microbiology profiles and in multidrug resistant pathogens prevalence. An analysis on the utility of the HCAI concept restricted to a single definition would be helpful to clarify this controversy.

The current review, restricted to all studies that used the definition proposed by Friedman et al. [1], intends to provide a systematic review on the microbiological profile involved in HCAI, to compare it with CAI and HAI and to evaluate the definition accuracy to predict infection by potentially drug resistant pathogens.

## Methods

A systematic review in accordance with PRISMA Statement was conducted. Objectives and methods of the study were specified in advance and documented in a protocol. This systematic review was registered at PROSPERO Systematic Review Registration with the Number CRD42014013648.

**Fig. 1:**
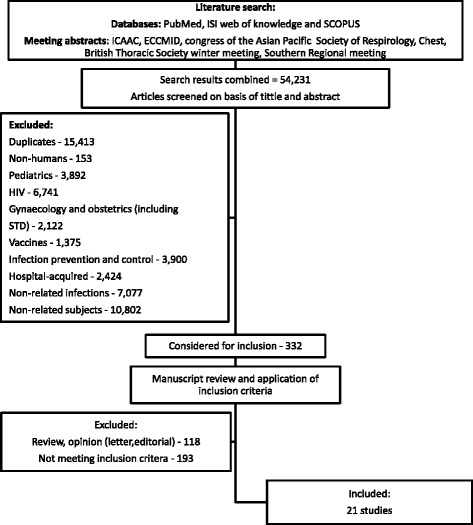
Selection of reports included in the analysis

The search was performed in accordance with the recommendations of the Cochrane collaboration, using MEDLINE/PubMed, SCOPUS and ISI Web of Knowledge, with the earliest achievable dating until January 2014. A manual search of references from reports, earlier reviews and retrieved studies was also performed. Books of abstracts and CD-ROMs from several annual scientific meetings were searched for relevant abstracts (Fig. 1). No language restrictions were applied and papers written in foreign languages were translated.

The electronic search strategy covered the main subject area: healthcare-associated infections (Additional file 1). The last search was performed on January 17^th^ 2014.

The inclusion criteria were all studies (excluding case series/reports) on adult patients admitted to hospital that used the definition of HCAI from Friedman et al. [1] and provided microbiology results by focus of infection, comparing with CAI and/or HAI.

The following classification of infection according to place of acquisition was used:

- CAI - infection detected within 48 h of hospital admission in patients without previous contact with healthcare services.
- HAI - localised or systemic condition: 1) that results from adverse reaction to the presence of an infectious agent(s) or its toxin(s) and 2) that was present 48 h or more after hospital admission and not incubating at hospital admission time [17].
- HCAI was defined according to the criteria proposed by Friedman et al. [1].

Potentially drug resistant (PDR) pathogens were defined as methicillin-resistant *Staphylococcus aureus, Pseudomonas* sp, *Acinectobacter* sp, *Stenotrophomonas maltophilia* and extended-spectrum beta-lactamases gram negative producers (ESBL) because these are usually not covered by the antibiotic therapy recommended for community-acquired infections [18–20].

The results of the literature search were accessed by two reviewers (TC, MA) and non-relevant studies, based on title and abstract, were excluded. For potentially relevant studies, the full text was obtained, and two investigators (TC, MA) independently assessed study eligibility and extracted data on study design, objectives of the included studies, microbiological profile by focus of infection and place of acquisition (HCAI, CAI and HAI) using a data extraction protocol; disagreements were resolved through consultation with a third reviewer (LA).

Each selected study was independently evaluated by two reviewers (TC, MA) in terms of strength of evidence through examination of the study design and quality of data.

Potential threats to the internal validity of included studies were evaluated considering the following criteria [21]:

- The authors define inclusion criteria;
- The authors define an adequate selection method (for instance, all hospitalized patients with infection);
- The selection of participants was consecutive;
- The outcome data was complete (microbiology data by place of acquisition) and reported (no attrition bias);
- All results were reported (reporting bias).

Studies that met all of the five criteria above were classified as “low risk of bias”. Studies that met one or more criteria only partially were classified as “moderate risk of bias”. Studies where one or more of these criteria was not met were classified as “high risk of bias”.

Detailed data on individual studies included in this review are provided in Additional file 2: Table S1. Data from the study by Cardoso et al. [22] is split in four components (respiratory, urinary, intra-abdominal and bloodstream infections), in order to facilitate microbiology profile interpretation and synthesis.

Odds ratios (ORs) comparing the incidence of PDR pathogens in HCAI *vs* CAI were calculated. Odds ratios were then pooled using a Mantel-Haenszel random effects model [23]. The sensitivity and specificity of the HCAI definition to predict infection by a PDR pathogen were calculated and the discriminative power studied through the analysis of the area under the summary receiver operator characteristics (sROC) curve [24]. Statistical heterogeneity was assessed using the Higgins *I*^*2*^ tests. Publication bias was assessed through the analysis of the funnel plot. Analyses were conducted using SPSS version 20, Review Manager 5 and Meta-DiSc for Windows.

## Results

The search retrieved a total of 54,231 references, among which 15,413 duplicates were identified. Of the 38,818 remaining articles, 38,486 were excluded based on title and abstract evaluation (Fig. 1). Full texts were obtained for the remaining 332 articles. Of these, 118 were review articles or opinion pieces (narrative reviews, commentaries and letters), 193 did not meet the inclusion criteria and 21 studies were included.

The description of the included studies by focus and place of acquisition of infection is shown in Additional file 2: Table S1, namely: pneumonia (Additional file 2: Table S1a), endocarditis (Additional file 2: Table S1b), urinary tract (Additional file 2: Table S1c), intra-abdominal (Additional file 2: Table S1d) and bloodstream infections (Additional file 2: Table S1e).

**Fig. 2:**
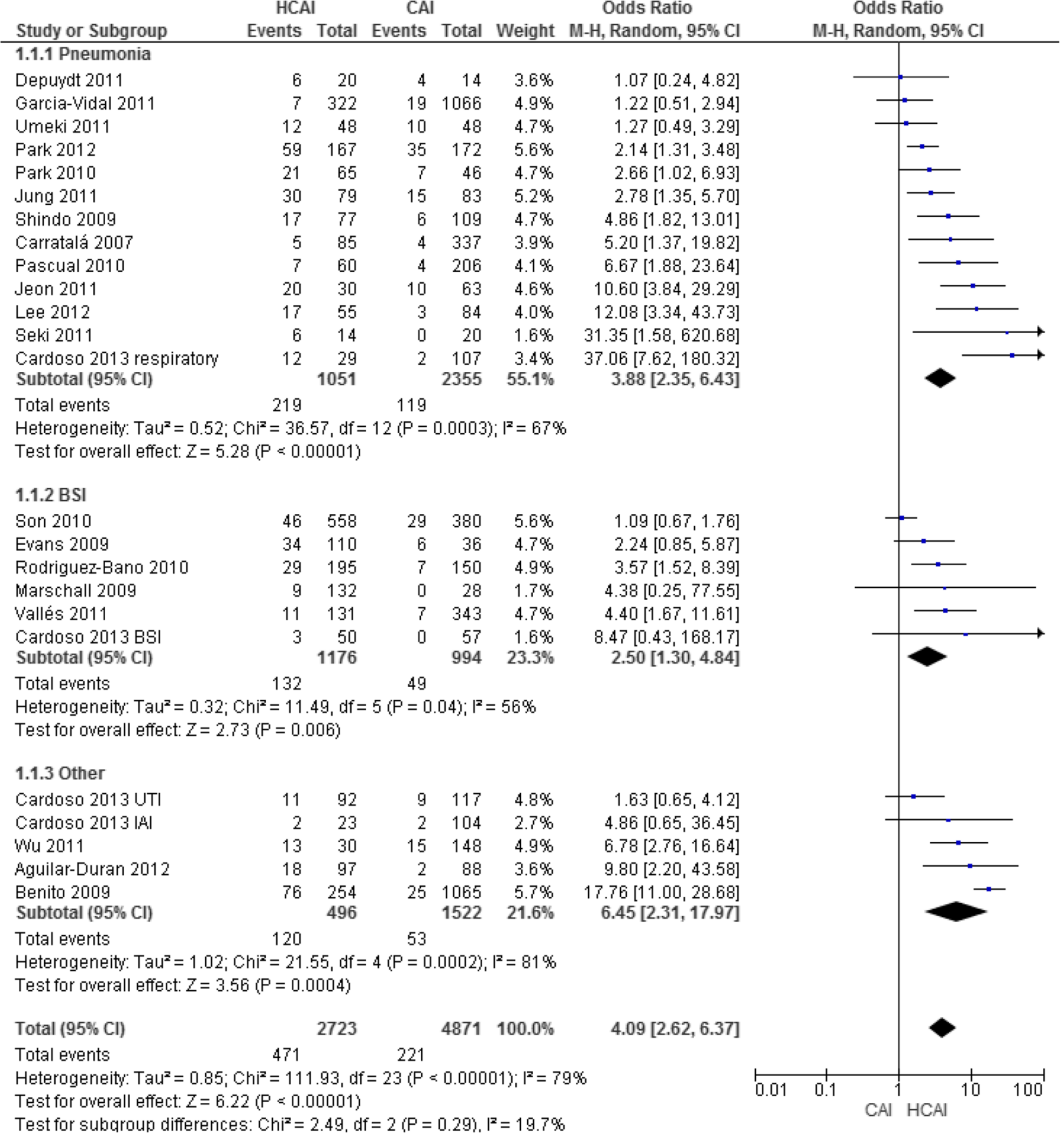
Association of healthcare-associated infection with infection by potentially drug resistant pathogens. Association of healthcare-associated infection with infection by potentially drug resistant pathogens when compared with community-acquired infection, for the main focus of infection. Abbreviations: CAI, community-acquired infection; HCAI, healthcare-associated infection; UTI, urinary tract infection; BSI, bloodstream infection; IAI, intra-abdominal infection; CI, confidence interval; M-H, Maentel-Haentzel; BSI, bloodstream infections

The 21 studies included for microbiology analysis involved a total of 12,096 patients of whom 3497 had HCAI, that in 2723 (78 % of cases) were microbiologically documented. Patients with HCAI represented 34 % of all patients admitted from the community (3497 among 10,300 patients). All studies included in the review show an association of HCAI with infection by a PDR pathogen that was significant in 15 (63 %) (Fig. 2). The pooled OR (95 % CI) was 4.09 (2.62–6.37), with significant heterogeneity (*I*^*2*^ = 79 %, *p* < 0.0001).

### Pneumonia

Healthcare-associated pneumonia (HCAP) was characterised in 1814 patients and of those 1051 had microbiological documentation (58 %) (Table 1): *Pseudomonas aeruginosa* was isolated in 102 (10 %), MRSA were isolated in 75 (7 %), ESBL strains in 18 (2 %), *Acinectobacter* spp in 8 (0.8 %) and *Stenotrophomonas maltophilia* in 6 (0.6 %) patients. In 129 (12.3 %) cases the authors did not specify the infectious agent.

**Table 1:** Microbiological profile of healthcare-associated, community and hospital-acquired infections by focus of infection

Pneumonia				Endocarditis			
Number of studies/patients	13 studies/6154 patients			Number of studies/patients	2 studies/1814 patients		
Number of patients (isolations)	HCAI - 1814 (1051–58 %)	CAI - 4264 (2355–55 %)	HAI - 147 (121–82 %)	Number of patients (isolations)	HCAI 284 (100 %)	CAI 1213 (100 %)	HAI 325 (100 %)
Microorganism, n (%)				Microorganism, n (%)			
*Streptococcus pneumoniae*	394 (37)	1389 (59)	18 (15)	Staphylococci	164 (59)	285 (24)	124 (38)
*Klebsiella pneumoniae*	114 (11)	70 (3)	6 (5)	MRSA (of all Staphylococci isolation)	89 (54)	39 (14)	58 (47)
*Staphylococcus aureus*	124 (12)	106 (4)	31 (26)	Enterococci	45 (16)	97 (8)	43 (13)
MRSA (of all Staphylococci isolation)	75 (60)	31 (29)	19 (61)	Coagulase negative Staphylococci	38 (14)	71 (6)	39 (12)
*Pseudomonas aeruginosa*	102 (10)	69 (3)	27 (22)	*Streptococcus viridans*	36 (13)	328 (27)	14 (4)
*Haemophilus influenzae*	56 (5)	179 (8)	1 (1)	Gram negative bacilli	0 (0)	2 (0.2)	1 (0.3)
Atypical agents	38 (4)	349 (15)					
*E. coli*	18 (2)	6 (0.3)	7 (6)				
PDR pathogens^a^, p value*	235 (22)	119 (5), *p* < 0.0001	56 (46), *p* < 0.0001	PDR pathogens^a^, p value*	89 (32)	40 (3), *p* < 0.0001	58 (18), *p* < 0.0001
Urinary tract infections				Bloodstream infection			
Number of studies/patients	2 studies/595 patients			Number of studies/patients	6 studies/3320 patients		
Number of patients (isolations)	HCAI 199 (189–95 %)	CAI 228 (205–90 %)	HAI 168 (163–97 %)	Number of patients	HCAI 1176	CAI 994	HAI 1337
Microorganism, n (%)				Microorganism, n (%)			
*E. coli*	120 (63)	147 (72)	61 (37)	*E. coli*	231 (20)	353 (35)	263 (20)
*Klebsiella spp*	17 (9)	12 (6)	17 (10)	*Staphylococcus aureus*	117 (10)	85 (9)	286 (21)
ESBL	18 (10)	4 (2)	9 (6)	MRSA (of all *Staphylococcus aureus isolation)*	42 (36)	10 (12)	186 (65)
*Pseudomonas aeruginosa*	10 (5)	5 (2)	19 (12)	*Klebsiella pneumoniae*	85 (7)	48 (5)	180 (13)
*Proteus spp.*	8 (4)	14 (7)	12 (7)	ESBL	21 (2)	14 (1)	61 (5)
*Other gram negative rods*	7 (4)	11 (5)	17 (10)	*Pseudomonas aeruginosa*	58 (5)	20 (2)	121 (9)
*Enterococcus spp.*	8 (4)	7 (3)	12 (7)	*Streptococcus pneumoniae*	21 (2)	132 (13)	15 (1)
PDR pathogens^a^, p value*	29 (15)	11 (5), *p* = 0.001	32 (20), *p* = 0.353	PDR pathogens^a^, p value*	132 (11)	49 (5), *p* < 0.0001	423 (32), *p* < 0.0001

*p value refers to comparison of HCAI with CAI or HCAI with HAI

^a^PDR pathogens = MRSA, *Pseudomonas* sp, *Acinectobacter* sp, *Stenotrophomonas maltophilia*, ESBL

**Fig. 3:**
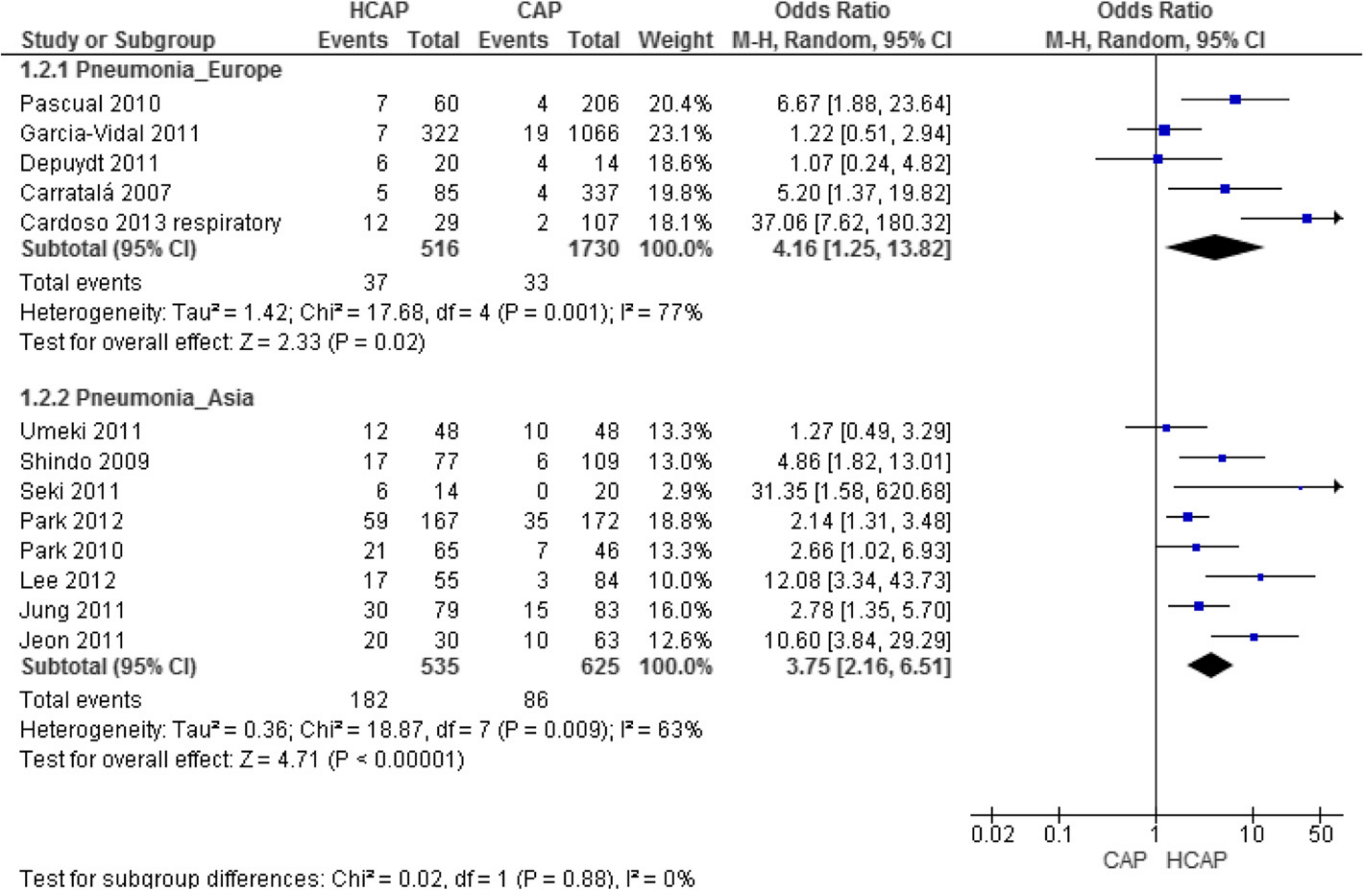
Association of HCAP with infection by PDR pathogens compared with CAP, according to geographic location. Abbreviations: CAP, Community-acquired pneumonia; HCAP, healthcare-associated pneumonia; PDR – potentially drug resistant; CI, confidence interval; M-H, Maentel-Haentzel

The prevalence of PDR pathogens was 21 % (*n* = 219) in HCAP, compared to 5 % (*n* = 119) in CAP (*p* < 0.001) and 46 % (*n* = 56) in HAP (*p* < 0.001). All studies show the association of HCAP with infection by a PDR pathogen (Fig. 3), that is significant in 10 (77 %). The pooled OR (95 % CI) was 3.88 (2.35–6.43), with high heterogeneity (*I*^*2*^ = 67 %, *p* = 0.003).

European studies [10, 22, 25–27], included 3894 patients: 815 in the HCAP group with an isolation rate of 83 % (*n* = 679), 2878 in the CAP group with an isolation rate of 60 % (*n* = 1732) and 147 in the HAP group with an isolation rate of 82 % (*n* = 121). The prevalence of PDR pathogens was 5 % (*n* = 37) in HCAP, compared to 2 % (*n* = 33) in CAP (*p* < 0.001) and 46 % (*n* = 56) in HAP (*p* < 0.001). The pooled OR (95 % CI) for infection by a PDR organism in the HCAP group was 4.16 (1.25–13.82), with important heterogeneity (*I*^*2*^ = 77 %, *p* = 0.001) (Fig. 3).

The remaining eight studies were from Asia and included 2331 patients: 945 in the HCAP group with an isolation rate of 57 % (*n* = 535) and 1386 in the CAP group with an isolation rate of 45 % (*n* = 625). The prevalence of PDR pathogens was 34 % (*n* = 182) in HCAP and 14 % (*n* = 86) in CAP (*p* < 0.001). The pooled OR (95 % CI) for association of HCAP and infection by a PDR pathogen was 3.75 (2.16–6.51), again with an important heterogeneity (*I*^*2*^ = 63 %, *p* = 0.009) (Fig. 3).

Focusing only in “low risk of bias” studies, seven studies including 4522 patients were analysed: 1120 in the HCAP group with an isolation rate of 68 % and 3297 in the CAP group with an isolation rate of 60 %, the prevalence of different microorganisms remained the same (with a maximum variation of 2 % in each), with the exception of PDR pathogens prevalence that decreased to 18 %, probably due to the higher rate of overall isolation. In this group six out of the seven studies showed a significant association of HCAP with infection by a PDR pathogen. The pooled OR (95 % CI) for infection by a PDR in the HCAP group was 5.35 (2.44–11.75), with *I*^*2*^ = 78 % (*p* = 0.0002).

The ATS/IDSA guidelines for the treatment of community-acquired pneumonia [18] recommend cefotaxime, ceftriaxone or ampicillin/sulbactam plus azithromycin or monotherapy with a fluroquinolone as an empirical antibiotic therapy. Considering only the species described among all isolations in HCAP (941 out of 1058), 711 (76 %) of the patients with HCAP would receive appropriate antibiotic therapy if the antibiotic therapy option is a fluroquinolone or a 3rd generation cephalosporins plus macrolide, if a beta-lactamic plus azithromycin was to be given the number would decrease to 691 (73 %) due to some gram negative uncovering.

The ATS/IDSA guidelines for the treatment of healthcare-associated pneumonia and hospital-acquired pneumonia [13] recommend as an empirical antibiotic therapy: cefepime, ceftazidime, imipenem, meropenem or piperacillin-tazobactam plus ciprofloxacin, levofloxacin, amikacin, gentamicin or tobramycin plus linezolid or vancomycin. If these recommendations are applied to the group of 941 patients included in this review, with microbiologically documented HCAP and infectious agent specified, 894 (95 %) would receive appropriate antibiotic therapy. Patients with *Acinectobater* spp. (*n* = 8) would only be covered if the isolated rods are sensitive, ESBL (*n* = 18) strains only if carbapenems are used and atypical agents (*n* = 38) if a fluroquinolone is given. *Stenotrophomonas* spp. (*n* = 6) and fungi (*n* = 3) would be left out regardless of either option. The use of these guidelines would result in overtreatment of at least 691 patients (73 %).

If piperacillin-tazobactam or aztreonam plus azithromycin were used to treat the same group of HCAP patients the rate for an appropriate antibiotic therapy would increase to 90 % (*n* = 843).

### Other foci of infection

There were only two studies on endocarditis: a small single centre study at an oncology department classified as “moderate risk of bias” [28] and a large, single centre, “low risk of bias” study [5] and both found significant differences in the microbiological profile between HCAI, CAI and HAI, particularly regarding the prevalence of PDR pathogens (Additional file 2: Table S1b, Table 1 and Fig. 2).

Two studies addressed urinary tract infection, both originating from small, single centres [6, 22], and described different microbiological profiles according to place of acquisition, but showing no significant differences in the prevalence of PDR pathogens by place of acquisition of infection (Additional file 2: Table S1c, Table 1 and Fig. 2).

One small single centre study described the microbiological profile of intra-abdominal infections [22], including significant differences between the microbiological profile of HCAI and CAI (*p* = 0.015), and HCAI and HAI (*p* = 0.008), but included only a small number of patients with microbiologically documented infection (Additional file 2: Table S1d and Fig. 2).

Bloodstream HCAI also showed a microbiological profile different between CAI and HAI (Additional file 2: Table S1e, Table 1 and Fig. 2). The proportion of PDR pathogens was 5 % in CAI, 11 % in HCAI and 32 % in HAI (*p* < 0.001). Among the included studies, none provided a microbiological description of primary bacteremia. The pooled OR (95 % CI) for infection by a PDR pathogen, in bloodstream infections, was 2.5 (1.3–4.8) for HCAI when compared to CAI (*I*^*2*^ = 56 %, *p* = 0.04) (Fig. 2).

### Accuracy of HCAI definition to predict infection by a PDR pathogen

**Fig. 4:**
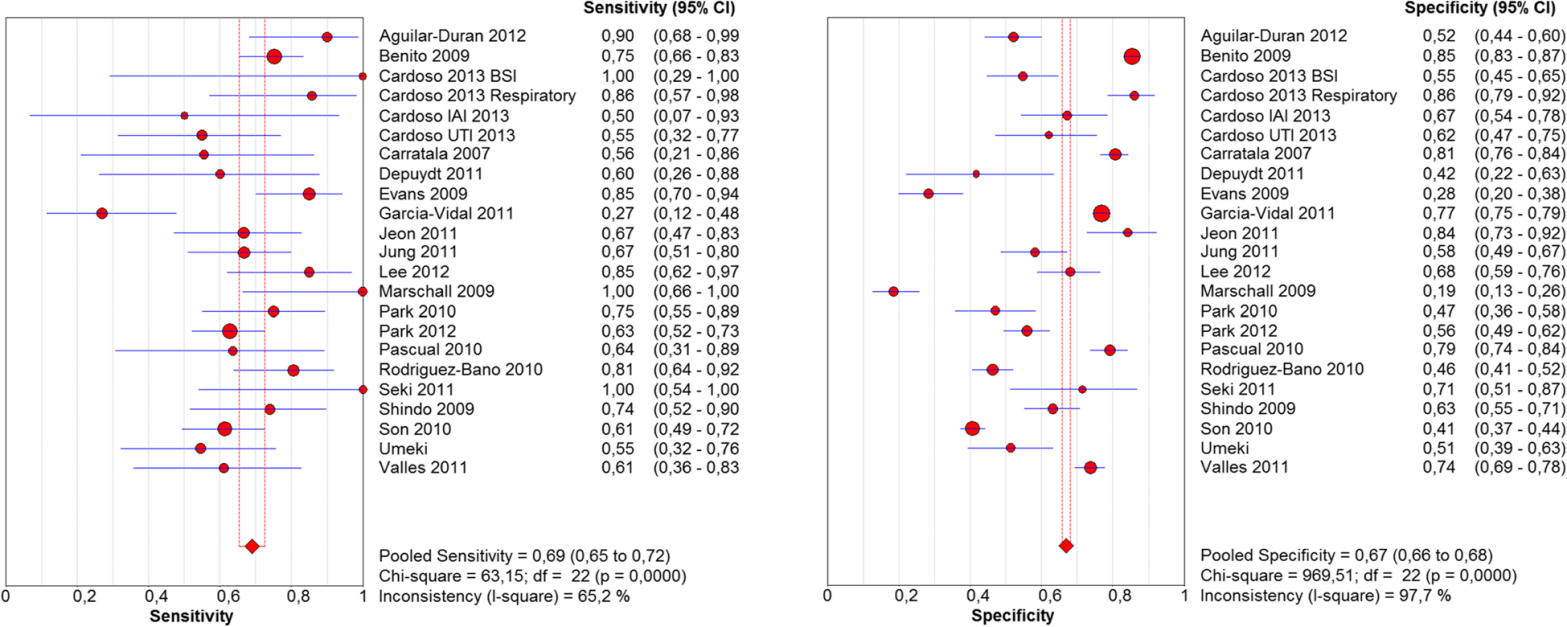
Sensitivity and specificity of HCAI definition to predict infection by potentially drug resistant pathogens. Abbreviations: CI, confidence interval; HCAI, healthcare-associated infection

The overall sensitivity of HCAI definition to predict infection by a PDR pathogen was 0.69 (0.65–0.72) and the associated specificity was 0.67 (0.66–0.68), with an *I*^*2*^*test* of 65 and 98 % respectively (Fig. 4).

**Fig. 5:**
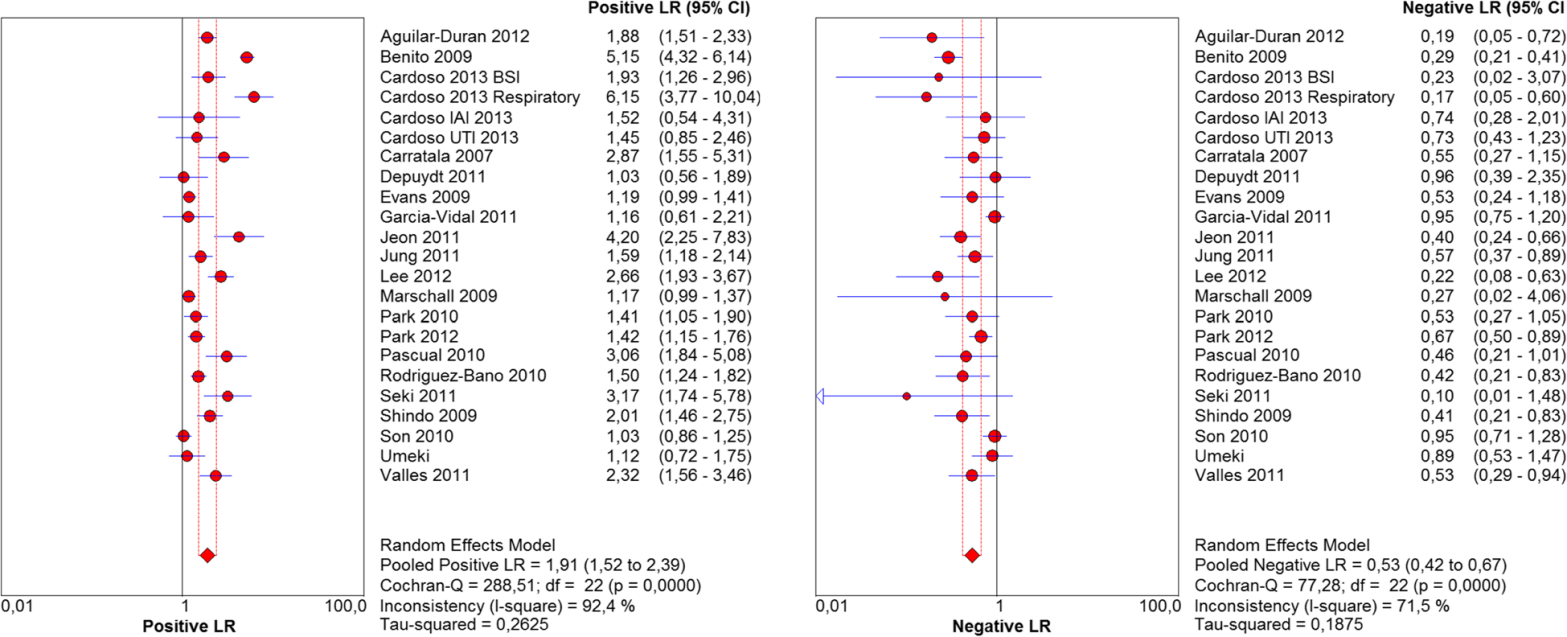
Positive and negative likelihood ratios (LR) for each study included in the analysis. Abbreviations: CI, confidence interval; LR, likelihood ratio

The pooled positive likelihood ratio (LR) was 1,9 and the pooled negative LR was 0,53, with an *I*^*2*^ test of 92 and 72 %, respectively (Fig. 5).

**Fig. 6:**
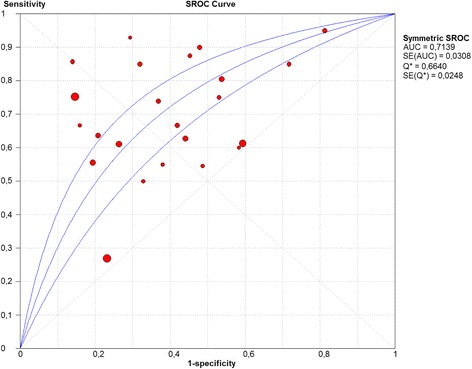
Discriminative power of HCAI definition to predict infection by PDR pathogens. Abbreviations: HCAI, healthcare-associated infection; PDR, potentially drug resistant; ROC – receiver operating curve

The area under the summary ROC curve was 0.71 (Fig. 6).

### Risk of bias

Of the included studies, eight presented “low”, four “moderate” and eight “high risk of bias” (Additional file 3: Table S2).

The analysis of the funnel plot shows asymmetry in the lower left quadrant. This may reflect a lack of small studies demonstrating a negative association of HCAI and infection by PDR pathogens or eventually an overrepresentation of positive studies.

## Discussion

HCAI represented more than a third of all patients admitted from the community setting into hospital care. Patients with HCAI presented different microbiological profiles when compared with community and hospital-acquired infections for the major foci of infection: respiratory, endocarditis, urinary, intra-abdominal and bloodstream infections.

Unfortunately only for pneumonia a fair number of epidemiological studies were found allowing a very good description of the microbiological profile involved, which is the only HCAI for which specific treatment recommendations are available [13] and they recommend broad spectrum antibiotics with an empiric coverage of *Pseudomonas* spp. and MRSA. In this review, *Pseudomonas aeruginosa* was present in only 10 % and MRSA in 7 % of all microbiologically documented HCAP. If these guidelines were used in this group of more than 1000 patients, over 70 % would receive overtreatment, which is a well-known factor for the development and increasing rate of multidrug resistance [29]. On the other hand, the application of CAP treatment guidelines would guarantee adequate antibiotic therapy in only 73 % of the patients with microbiologic documentation of infection. The use of piperacillin-tazobactam or aztreonam plus azithromycin would increase the rate of adequate antibiotic therapy to 90 %, without creating such large unnecessary microbiologic pressure.

It is possible that a narrower antibiotic spectrum might be appropriate in specific groups of patients, such as those without severe sepsis/septic shock and those without immunosuppression, but the studies included in this review did not provide data to address these items. Nursing home-acquired (NHAP) pneumonia is the subgroup of HCAI more studied: patients with non-severe disease have a pathogen distribution similar to those expected in CAP [30] and among patients with severe NHAP (that is with organ dysfunction), resistant pathogens have been seen [10, 30, 31], suggesting that severity of acute illness plays a role in the MDR prevalence. The use of CAP guidelines treatment in non-severe NHAP is supported by the study by El Solh et al. [32] that reported no impact in clinical outcomes between those patients treated according to the HCAP vs. the CAP algorithm. These findings are also supported by the study from Labelle et al. [33] where the use of CAP guidelines to treat culture negative non-severe HCAP was shown to be successful and the study by Attridge et al. [34] where the treatment of non-severe HCAP patients, with guideline concordant HCAP therapy was not associated with improved survival compared with guideline concordant CAP therapy (in fact it was associated with increased mortality). Thus, if one starts with a broad spectrum HCAP regimen and by day 3 no microbiologic data exist to facilitate de-escalation clinicians can feel comfortable narrowing the antimicrobial therapy to no more than a CAP like regimen, particularly in non-severe HCAP.In endocarditis, two studies were analysed [5, 28]: the first one included few patients and had a “moderate risk of bias”; the second one [5] included a significant number of patients but the exclusion of intra-venous drug users and patients with prosthetic valves probably deviated the predicted profile to show a lower rate of *Staphylococci* spp., including MRSA, thus preventing generalisation of the findings.

Regarding urinary tract and intra-abdominal infections the inclusion of few, small studies with few patients, prevents firm conclusions and generalisation. Clearly larger, multicentre studies, designed towards microbiological characterisation are needed.

The six studies on bloodstream infections gathered a considerable number of patients, with similar proportions of patients in the three groups. However, in order to discuss empiric antibiotic therapy, it is necessary to consider the microbiological profile of primary BSI and none of the studies mention this. In fact only two studies [22, 35] described the proportion of primary BSI by place of acquisition, but still without describing the microorganisms involved.

The lack of good quality studies regarding different focus apart from pneumonia prevented an accurate description of the expected microbiologic profile for each of the major focus of infection. Further studies on endocarditis, urinary, intra-abdominal and bloodstream infections are needed focusing on detailed microbiologic profile involved in CAI, HCAI and HAI, to confirm the utility of maintaining HCAI category to influence antibiotic prescription.

All studies included in this review used Deborah Friedman’s HCAI definition [1] and showed an association of HCAI with infection by a PDR organism that was significant in the vast majority. Unfortunately, interpretation of the summary measures was not possible due to the associated medium-high heterogeneity (*I*^*2*^*test* > 50 %). The discrimination power of the adopted HCAI definition to predict infection by a PDR pathogen was fair, translated by an AU-sROC curve of 0.71 (Fig. 6), which is in accordance with a recent systematic review and meta-analysis on the accuracy of HCAP definition to identify potentially resistant pathogens [15]. The HCAP definition adopted by the authors was the one proposed by ATS/IDSA [13], but only five of the included studies met that definition [24, 31, 36–38]; the additional 19 studies used different definitions (some of them included immunosuppression) [7, 39] and the majority [10, 25, 26, 40–45] used the definition proposed by Friedman et al. [1]. The nine studies that used the definition by Friedman et al. [1] were also included in the current review. Again in that review all studies included showed an association of HCAP with infection by a PDR pathogen.

In the 2011 European guidelines on adult respiratory tract infections [16], the task force stated that although the term HCAP has been putted forward after the 2005 publication of the ATS guidelines [13], evidence base did not support the use of this term as being clinically relevant in Europe at the time. The task force pointed 15 studies as evidence for this statement: two are part of the current systematic review [10, 43], two used different definitions of HCAP [7, 46], seven were on nursing home community-acquired pneumonia [47–53], one on *Staphylococcus aureus* pneumonia [54] and the rest did not present any data on microbiology and/or comparison with CAP microbiology [55–57]. The studies mentioned in the guidelines that used a clear definition of HCAP and compare the microbiology findings with CAP all show an association of HCAP definition with infection by a PDR pathogen [7, 10, 43, 46], which does not support the initial statement of the task force.

The results of the current review reinforce the association of HCAI definition with infection by PDR pathogens, the fact that it is not translated in good discrimination power could be explained by the high heterogeneity of the studies included in the review. Also the diverse criteria included in the definition might explain part of this heterogeneity. A study focusing on the individual components of HCAI definitions is needed to determine the associated weight of each risk factor with an infection by a MDR pathogen, to further stratify patients in different levels of risk of infection by a MDR pathogen, prompting different antibiotic recommendations according to the predetermined risk, saving broad spectrum antibiotic schemes for those patients with high risk, and treating the rest with the narrower spectrum possible, which will probably be the same as CAP for the low risk patients.

The prevalence of PDR pathogens among patients admitted to the hospital, from the community, with pneumonia (either CAP or HCAP) is higher in Asia, but the association of infection by a PDR pathogen with HCAP (measured by the OR) was similar to that found in Europe. This association was independent of quality of included studies (either overall or considering only “low risk of bias” studies) reinforcing the maintenance of the three categories: community, healthcare-associated and hospital-acquired pneumonia, in its classification.

This review encountered several limitations. Firstly retrospective cohort studies had to be included, which might have limited the quality of clinical and microbiological data and information.

Secondly, the rightful use of a strict case definition allowed pooling of the results from different studies, however the wide heterogeneity in the definitions of HCAI used in published studies led to the exclusion of many studies from other parts of the world that might have shown different results. The HCAI definition [1] chosen followed the findings of a recent systematic review [3] that show that it is the most frequently used definition in clinical research.

Thirdly, substantial heterogeneity between studies was detected, which prevents the strict adoption of the summary measure; this heterogeneity was independent of the geographic area, overall quality of studies and focus of infection. Using a more homogeneous population would solve this, but would cause selection bias.

Fourthly, publication bias was detected. Studies with positive findings are more likely to be published, but after having researched references from reports, abstract books and CD-ROMs from related scientific meetings, places where negative findings are more likely to be reported, it was concluded that these might have been reported, but never published.

Fifth, despite the exhaustive bibliographic search described and preformed there is always the possibility of missing studies that could have met the inclusion criteria.

This review also has several strengths: the research method was exhaustive, leading to a huge number of retrieved studies and a very long process of selection; this type of sensitive electronic search was conducted in several databases, including relevant conference proceedings and a manual search of additional sources, which ensures that no relevant study was excluded.

The permissive criteria for study inclusion in this review were essential to achieve the main goal: gathering all studies on HCAI that provided a detailed microbiology profile by focus of infection.

The careful selection of studies using exactly the same definition of HCAI, CAI and HAI minimises the bias related to overlapping categories and allows a proper comparison between studies and summing of the results found. This has been the major limitation of other studies and consensus, which have used and pooled results of studies using an heterogeneous set of definitions that preclude and bias pooled results and conclusions [15, 16].

The isolation rate was high (78 %), that is partially due to the focus of infection included in the analysis: endocarditis and bloodstream infection that are dependent on microbiology identification for diagnosis and urinary tract infection that are also highly dependent on microbiologic documentation; even in HCAP microbiologic documentation of infection was possible in more than half of the cases, so we believe that the described profile are representative of the all cohort.

The majority of included studies were considered of moderate or high quality considering the simplicity of the evaluation. The criteria used were based on the STROBE Statement: clear definition of inclusion criteria and selection method, consecutive selection of patients, and no attrition or reporting bias. Considering the found of observational studies the researchers think that those are the most adequate criteria to evaluate risk of bias in this type of studies. Although this is a very relevant analysis, the fact is that we found no major differences among studies depending on their methodological quality.

## Conclusions

This systematic review provides evidence that HCAI is significantly different from CAI and HAI, representing a separate group of infections in terms of the microbiology profile, including a significant association with infection by PDR pathogens, for the main focus of infection.

An accurate microbiological characterization of HCAI was not possible for the main focus of infection apart from pneumonia due to the lack of good epidemiological studies in this area.

The accuracy of Friedman et al. HCAI definition for predicting infection by PDR pathogens was fair, but all studies included showed a consistent association of HCAI with infection by PDR.

The pursue of good epidemiological studies is of the utmost relevance to allow a good microbiological characterization of HCAI for the main focus of infection helping the clinician in the selection of empiric antibiotic therapy and international societies in the development of specific treatment recommendations.

## Additional files

Additional file 1:
**Search strategy details.** (DOCX 14 kb) Additional file 2: Table S1. List of included studies describing the microbiologic profile by foci and place of acquisition of infection. (DOCX 70 kb) Additional file 3: Table S2. Studies with moderate or high risk of bias according to pre-defined criteria. (DOCX 36 kb)

## Abbreviations

ATS/IDSA: American Thoracic Society/Infectious Diseases Society of America
AU-sROC: area under the sROC
BSI: bloodstream infection
CAI: community-acquired infection
CI: confidence interval
ESBL: extended-spectrum β-lactamases
HCAI: healthcare-associated infection
HAI: hospital-acquired infection
HCAP: healthcare-associated pneumonia
LR: likelihood ratio
MRSA: methicillin-resistant *Staphylococcus aureus*
OR: odds ratio
PDR: potentially drug resistant pathogens
sROC: summary receiver operating characteristics
