# Supplementary material for: Microbiology of healthcare-associated infections and the definition accuracy to predict infection by potentially drug resistant pathogens: a systematic review
Source: BMC Infect Dis. 2015 Dec 11;15:565. doi: 10.1186/s12879-015-1304-2 (PMC4676854; doi:10.1186/s12879-015-1304-2)
Supplement: Additional file 2: Table S1. — List of included studies describing the microbiologic profile by foci and place of acquisition of infection. (DOCX 70 kb) [file 12879_2015_1304_MOESM2_ESM.docx]

**Table S1. – List of included studies describing the microbiologic profile by foci and place of acquisition of infection**

**a )Pneumonias**

| Study | Umeki, 2011 [^1^](#_ENREF_1) | | Park, 2012 [^2^](#_ENREF_2) | | Shindo, 2009 [^3^](#_ENREF_3) | | Seki, 2011 [^4^](#_ENREF_4) | | Garcia-Vidal,2011 [^5^](#_ENREF_5) | | Jung, 2011 [^6^](#_ENREF_6) | | Jeon,2011 [^7^](#_ENREF_7) | |
| --- | --- | --- | --- | --- | --- | --- | --- | --- | --- | --- | --- | --- | --- | --- |
| Design | Prospective, single centre study, in 202 patients, Japan, 2 years | | Prospective, single centre study, in 339 patients with positive microbiology, Korea, 2 years | | Retrospective, single centre study, in 371 patients, Japan, 1 year and 3 months | | Retrospective, single centre study, in 34 patients, Japan, 4 months | | Prospective, single centre study, in 2153 patients, Spain, 8 years and 9 months. Patients having more than one condition of HCAI, with neutropenia, AIDS, after transplantation and chronic corticosteroid treatment were excluded | | Retrospective, single centre study, in 527 patients, Korea, 1 year | | Retrospective, multicentre study, in 210 patients older than 60 years, Korea, 2 years | |
| Patients in each category, n(%) | HCAI  79(39) | CAI  123 (61) | HCAI  167 (49) | CAI  172 (51) | HCAI  141(38) | CAI  230 (62) | HCAI  14 (41) | CAI  20 (59) | HCAI  485 (26) | CAI  1668 (74) | HCAI  231(44) | CAI  296 (56) | HCAI  35 (17) | CAI  175 (83) |
| Isolation rate,n(%) | 48(61) | 48 (39) | 167(100) | 172(100) | 77(55) | 109 (47) | 14(100) | 20(100) | 322 (66) | 1066 (64) | 79(34) | 83 (28) | 30(86) | 63 (36) |
| *Streptococcus pneumoniae,*n(%) | 12(25) | 12 (25) | 22 (13) | 55 (32) | 19 (25) | 44(40) | 3 (21) | 13 (65) | 188(58) | 686 (64) | 11(14) | 33 (40) | 2 (7) | 21 (33) |
| *Haemophilus influenzae*, n(%) | 2 (4) | 10 (21) | 2 (1) | 4 (2) | 4 (5) | 17 (16) | 0 (0) | 2 (10) | 25 (8) | 80 (8) | 2 (3) | 3 (4) | 1 (3) | 7 (11) |
| *Klebsiella pneumoniae*,n(%) | 8 (17) | 1 (2) | 45 (27) | 26 (15) | 10 (13) | 4 (4) | 1 (7) | 0 (0) |  |  | 23 (29) | 13 (16) | 8 (27) | 13 (21) |
| *Pseudomonas aeruginosa,* n(%) | 2 (4) | 4 (8) | 35 (21) | 23 (13) | 8 (10) | 4 (4) | 3 (21) | 0 (0) | 7 (2) | 19 (2) | 10 (13) | 5 (6) | 8 (27) | 4 (6) |
| *Staphylococcus aureus*, n(%) | 12 (25) | 4 (8) | 29 (17) | 24 (14) | 14 (13) | 14(18) | 3 (21) | 1 (8) | 6 (2) | 5 (0.5) | 13(16) | 15 (18) | 12 (40) | 9 (11) |
| MRSA (of all *Staphylococcus aureus*), n(%) | 9 (75) | 4 (100) | 18(62) | 9 (38) | 5 (5) | 2 (3) | 3(100) | 0(0) |  |  | 9 (69) | 6 (40) | 9 (75) | 6(66) |
| *E. coli,* n(%) | 1(2) | 1(2) | 5 (3) | 2 (1) | 0(0) | 1 (1) | 0(0) | 0(0) |  |  | 1(1) | 0(0) |  |  |
| Atypical agents, n(%) | 2(4) | 7(15) | 5 (3) | 27 (16) | 1(1) | 16 (21) | 0 (0) | 1 (8) | 21(7) | 192 (18) | 0 (0) | 3 (4) | 0 (0) | 2 (3) |
| PDR^#^, n(%)  p value* | 12 (25) | 10 (21)  p=0.627 | 59 (35) | 35 (20)  p=0.002 | 17 (22) | 6 (6)  p=0.001 | 6 (43) | 0 (0)  p=0.002 | 7 (2) | 19 (2)  p=0.650 | 30 (38) | 15 (18)  p=0.005 | 20 (67) | 10 (16)  p<0.0001 |
| Risk of bias | High | | Low | | Low | | High | | Low | | Moderate | | Low | |

*p value refers to comparison of HCAI with CAI or HCAI with HAI

**a) Pneumonias (continuation)**

| Study | Cardoso, 2013 [^8^](#_ENREF_8) | | | Lee, 2012 [^9^](#_ENREF_9) | | Depuydt, 2011 [^10^](#_ENREF_10) | | Pascual, 2010 [^11^](#_ENREF_11) | | | Park, 2010 [^12^](#_ENREF_12) | | Carratalà, 2007 [^13^](#_ENREF_13) | |
| --- | --- | --- | --- | --- | --- | --- | --- | --- | --- | --- | --- | --- | --- | --- |
| Design | Prospective, single centre study, in 1035 patients (419 with pneumonia), Portugal, 1 year | | | Retrospective, multicentre study, in 303 patients, Korea, 21 months | | Retrospective, single centre study, 287 episodes in 269 patients, Belgium, 1 year. Patients with neutropenia, transplantation or transferred from another hospital were excluded | | Retrospective, single centre study, in 308 patients with bacteriemic pneumonia, Spain , 6 years | | | Retrospective, single centre study, in 345 patients, Korea, 1 year. Defines CAI and HCAI until 72 h after hospital admission. Patients with neutropenia, AIDS and after transplantation were excluded | | Prospective, single centre study, in 727 patients, Spain, 4 years. Patients with neutropenia, AIDS and after transplantation were excluded | |
| Patients in each category, n (%) | HCAI  70(17) | CAI  244(58) | HAI  105 (25) | HCAI  96 (32) | CAI  207 (68) | HCAI  128 (45) | CAI  159 (55) | HCAI  60 (20) | CAI  206 (67) | HAI  42 (13) | HCAI  182 (53) | CAI  163 (47) | HCAI  126  (17) | CAI  601  (83) |
| Isolation rate,n(%) | 29(41) | 107(44) | 79 (75) | 55 (57) | 84 (41) | 20 (16) | 14 (9) | 60(100) | 206 (100) | 42(100) | 65 (36) | 46 (28) | 85 (67) | 337 (56) |
| *Streptococcus pneumoniae*, n(%) | 5 (17) | 58 (54) | 1 (1) | 24 (44) | 41 (49) | 6 (30) | 9 (64) | 42 (70) | 185 (90) | 17 (40) | 25 (38) | 28 (61) | 35(41) | 204(61) |
| *Haemophilus influenzae*, n(%) | 1 (3) | 14 (13) | 1 (1) | 0 (0) | 3 (4) |  |  |  |  |  | 4 (6) | 2 (4) | 15 (18) | 36 (11) |
| *Klebsiella pneumoniae*, n(%) | 1 (3) | 5 (5) | 6 (8) | 5 (9) | 3 (4) |  |  |  |  |  | 13 (20) | 4 (9) | 0(0) | 1 (0.3) |
| *Pseudomonas aeruginosa*, n(%) | 4 (14) | 2 (2) | 14 (18) | 4 (7) | 1 (1) |  |  | 7 (12) | 4 (2) | 13 (31) | 9 (14) | 2 (4) | 5 (6) | 3 (1) |
| *Staphylococcus aureus*, n(%) | 8 (28) | 5 (5) | 27 (24) | 9 (16) | 5 (6) | 3 (15) | 13 (93) | 2 (3.3) | 2 (1) | 4 (9.5) | 13 (20) | 6 (13) | 0(0) | 3 (1) |
| MRSA (of all *Staphylococcus aureus*), n(%) | 7 (88) | 0 (0) | 19 (70) | 8(89) | 2(40) | 2 (37) | 0 (0) |  |  |  | 5 (38) | 1(17) | 0(0) | 1 (33) |
| E. coli, n(%) | 3 (10) | 0 (0) | 7 (9) | 2 (4) | 0 (0) |  |  |  |  |  | 3 (5) | 0 (0) | 3 (4) | 2 (0.6) |
| Atypical agents, n(%) | 0 (0) | 8 (7) | 0 (0) | 4 (7) | 18 (21) |  |  |  |  |  |  |  | 5 (6) | 75 (22) |
| PDR^#^, n(%)  p value* | 12(41) | 2 (2)  p<0.0001 | 43 (54)  p=0.229 | 17 (31) | 3 (4)  p<0.0001 | 6 (30) | 4 (29)  p>0.999 | 7 (12) | 4 (2)  p=0.003 | 13 (31)  p=0.016 | 21 (32) | 7 (15)  p=0.041 | 5 (6) | 4 (1)  p=0.019 |
| Risk of bias | Low | | | Low | | High | | High | | | Moderate | | Low | |

*p value refers to comparison of HCAI with CAI or HCAI with HAI

**b) Endocarditis**

| Study | Wu, 2011 [^14^](#_ENREF_14) | | | Benito, 2009 [^15^](#_ENREF_15) | | |
| --- | --- | --- | --- | --- | --- | --- |
| Design | Retrospective, single centre study, in a tertiary care centre with a large oncology department, including 190 episodes in 192 patients, Taiwan, 5 years | | | Prospective, single centre study, in 1622 patients, USA, 6 months. Intra-venous drug user, prosthetic valves were excluded. | | |
| Number of patients per category, n (%) | HCAI  30 (15) | CAI  148  (74) | HAI  22 (11) | HCAI  254 (53) | CAI  1065  (11) | HAI  303  (36) |
| Staphylococci, n(%) | 23(77) | 75 (51) | 17(77) | 141 (59) | 210 (22) | 107 (37) |
| MSSA (of all Staphylococci isolation), n(%) | 8(35) | 57(76) | 0(0) |  |  |  |
| MRSA (of all Staphylococci isolation), n(%) | 13(57) | 14(19) | 17(100) | 76 (32) | 25(12) | 41(14) |
| Coagulase negative Staphylococci, n(%) | 2(9) | 4(5) | 0(0) | 36(15) | 67(7) | 39(14) |
| Streptococci, n(%) | 4(13) | 51 (34) | 2(9) |  |  |  |
| *Streptococcus viridans,* n(%) | 3(75) | 35(24) | 0(0) | 33(14) | 293 (31) | 14(5) |
| Enterococci, n(%) | 3(10) | 5(3) | 1(5) | 42(18) | 92 (10) | 42 (15) |
| Gram negative bacilli, n(%) | 0(0) | 2(1) | 1(5) |  |  |  |
| PDR^#^, n(%)  p value* | 13(43) | 15(10)  p<0.0001 | 17(77)  p=0.417 | 76(30) | 25(2)  p<0.0001 | 41(14)  p<0.0001 |
| Risk of bias | Moderate | | | Low | | |

*p value refers to comparison of CAI with healthcare associated = non nosocomial + nosocomial and HCAI with nosocomial

**c) Urinary tract infection**

| Study | Aguilar-Duran, 2012 [^16^](#_ENREF_16) | | | Cardoso, 2013 [^8^](#_ENREF_8) | | |
| --- | --- | --- | --- | --- | --- | --- |
| Design | Prospective, single centre study, in 251 patients with positive cultures, Spain, 7-8 months | | | Prospective, single centre study, 1035 patients (344 with UTI), Portugal, 1 year | | |
| Number of patients per category, n (%) | HCAI  97 (13) | CAI  88 (35) | HAI  66 (26) | HCAI  102 (30) | CAI  140 (40) | HAI  102 (30) |
| Isolation rate,n(%) | 97 (100) | 88 (100) | 66 (100) | 92 (90) | 117 (84) | 97 (95) |
| *E. coli*, n(%) | 69 (71) | 66 (75) | 36 (55) | 51 (56) | 81 (69) | 25 (26) |
| *Klebsiella* spp, n(%) | 8 (8) | 9 (10) | 10 (15) | 9 (10) | 3 (3) | 7 (7) |
| ESBL | 9 (12) | 1 (1) | 6 (13) | 9 (10) | 3 (3) | 3 (3) |
| *Pseudomonas aeruginosa*, n(%) | 9 (9) | 1 (1) | 7 (11) | 1 (1) | 4 (3) | 12 (12) |
| *Proteus* spp, n(%) | 4 (4) | 5 (6) | 4 (6) | 8 (9) | 9 (8) | 8 (8) |
| Other gram negative rods, n(%) | 2 (2) | 4 (5) | 6 (9) | 7 (8) | 7 (6) | 12 (12) |
| *Enterococcus* spp, n(%) | 4 (4) | 1 (1) | 1(2) | 4 (4) | 8 (7) | 11 (11) |
| PDR^#^, n(%)  p value* | 18 (19) | 2 (3)  p=0.003 | 15 (23)  p=0.233 | 11 (12) | 9 (8)  p=0.298 | 17 (18)  p=0.281 |
| Risk of bias | High | | | Low | | |

*p value refers to comparison of HCAI with CAI or HCAI with HAI

**d) Intra-abdominal (IAI) infections**

| Study | Cardoso, 2013 [^8^](#_ENREF_8) | | |
| --- | --- | --- | --- |
| Design | Prospective, single centre study, 1035 patients (213 with IAI), Portugal, 1 year | | |
| Number of patients per category, n (%) | HCAI  31 (15) | CAI  104 (49) | HAI  78 (36) |
| Isolation rate,n(%) | 23 (74) | 45 (43) | 56 (72) |
| *E. coli*, n(%) | 4 (17) | 15 (33) | 10 (18) |
| *Klebsiella* spp, n(%) | 0 (0) | 2 (4) | 2 (4) |
| ESBL | 1 (4) | 1 (2) | 2 (4) |
| *Pseudomonas aeruginosa*, n(%) | 0 (0) | 0 (0) | 2 (4) |
| Other gram negative rods, n(%) | 4 (17) | 5 (11) | 0 (0) |
| *Enterococcus* faecium, n(%) | 0 (0) | 0 (0) | 3 (5) |
| *Clostridium difficile*, n(%) | 1 (4) | 0 (0) | 5 (9) |
| Fungi, *n(%)* | 0 (0) | 2 (4) | 4 (7) |
| PDR^#^, n(%)  p value* | 2 (9) | 2 (4),  p=0.599 | 8 (14),  p=1.000 |
| Risk of bias | Low | | |

*p value refers to comparison of HCAI with CAI or HCAI with HAI

**e )Bloodstream infections (BSI)**

| Study | Marschall, 2009 [^17^](#_ENREF_17) | | | Son, 2010 [^18^](#_ENREF_18) | | | Evans, 2009 [^19^](#_ENREF_19) | | | Rodriguez-Bano, 2010 [^20^](#_ENREF_20) | | | Vallés, 2011 [^21^](#_ENREF_21) | | | Cardoso, 2013 [^8^](#_ENREF_8) | | |
| --- | --- | --- | --- | --- | --- | --- | --- | --- | --- | --- | --- | --- | --- | --- | --- | --- | --- | --- |
| Design | Prospective, single centre study, in 250 patients with gram negative bacteremia, USA, 6 months | | | Prospective, multicentre study, in 1144 patients, Korea, 12 months | | | Retrospective. Multicentre study, in 226 patients with spinal cord injury and disorder, USA, 7 years. Analyse episodes and not patients | | | Prospective, multicentre study, in 821 isolates, Spain, 2-5 months. Included different episodes in the same patient and potential contaminants too | | | Prospective, multicentre, international, in 726 patients, Spain and Argentina, 12 months | | | Prospective, single centre study, 1035 patients (153 with BSI), Portugal, 1 year | | |
| Patients per category,n(%) | HCAI  132 (53) | CAI  28  (11) | HAI  90  (36) | HCAI  558  (49) | CAI  380  (33) | HAI  206  (18) | HCAI  110  (27) | CAI  36  (9) | HAI  267  (64) | HCAI  195  (24) | CAI  150  (18) | HAI  476  (58) | HCAI  131  (18) | CAI  343  (47) | HAI  252  (35) | HCAI  50  (33) | CAI  57  (37) | HAI  46  (30) |
| *E. coli*, n(%) | 33(25) | 16(57) | 10(11) | 56(27) | 179(47) | 83 (15) | 19(17) | 9(25) | 24(9) | 72(37) | 57 (38) | 96 (20) | 35(27) | 70 (21) | 46 (19) | 16 (32) | 22 (39) | 4(9) |
| *Klebsiella pneumoniae*,n(%) | 20(15) | 2(7) | 23(26) | 34 (17) | 12 (13) | 74 (13) | 10(9) | 6(17) | 26(10) | 13 (7) | 12 (8) | 39 (8) | 6 (5) | 13 (4) | 15(6) | 2 (4) | 3 (5) | 3(6) |
| *Pseudomonas aeruginosa*,n(%) | 9 (7) | 0(0) | 10(11) | 16 (8) | 11 (3) | 36(7) | 14(13) | 2(6) | 29(11) | 13 (7) | 1(<1) | 26 (5) | 6 (5) | 6 (2) | 16 (6) | 0 (0) | 0 (0) | 4(9) |
| *Staphylococcus aureus*, n(%) |  |  |  | 29 (14) | 28 (7) | 85 (15) | 33(30) | 12(33) | 97(36) | 22(11) | 10 (7) | 67 (14) | 18 (14) | 34 (10) | 25 (10) | 15(30) | 1 (2) | 12(26) |
| MRSA (of all *Staphylococcus aureus*), n(%) |  |  |  | 9 (31) | 5 (18) | 59 (69) | 20(18) | 4 (11) | 82 (31) | 6 (27) | 0 (0) | 24 (36) | 5(28) | 1 (3) | 13 (52) | 2 (13) | 0 (0) | 8(67) |
| *Streptococcus pneumoniae*,n(%) |  |  |  | 2 (1) | 15 (4) | 7 (1) |  |  |  | 11 (6) | 27 (18) | 3 (<1) | 6 (5) | 73 (21) | 4 (2) | 2 (4) | 17 (30) | 1(22) |
| ESBL, n(%) |  |  |  | 13(14) | 8 (4) | 40 (25) |  |  |  | 7 (8) | 6 (9) | 21 (16) |  |  |  | 1 (2) | 0 (0) | 0 (0) |
| PDR^#^, n(%)  p value* | 9 (7) | 0(0) | 10(11) p=0.262 | 46(8) | 29 (8)  p=0.734 | 162(79)  p<0.0001 | 34 (31) | 6 (17)  p=0.096 | 111(42)  p=0.053 | 29(15) | 7 (5)  p=0.002 | 99 (21)  p=0.076 | 11 (8) | 7 (2)  p=0.001 | 29(12)  p=0.345 | 3 (6) | 0 (0) | 12 (26)  P=0.01 |
| **Primary bacteremia,n(%)** | Not mentioned | | | HCAI,28  (5) | CAI, 22  (6) | HAI, 29  (14) | Not mentioned | | | Not mentioned | | | Not mentioned | | | HCAI 21(42) | CAI 4(7) | HAI 32(69) |
| **Focus of 2 ^ry^ bacteremia** | HCAI | CAI | HAI | HCAI | CAI | HAI |  | | | HCAI | CAI | HAI | HCAI | CAI | HAI | HCAI | CAI | HAI |
| Intravascular device | 26(20) | 0(0) | 14(16) |  |  |  |  | | | 24(12) | 0(0) | 116(24) | 10 (8) | 0 (0) | 37(15) | 17(34) | 0(0) | 22(48) |
| Urinary | 38(20) | 16(57) | 13(14) | 30(14) | 119(31) | 32(6) |  | | | 41(21) | 46 (31) | 73(15) | 23(18) | 40(12) | 16(7) | 22(44) | 21(37) | 5(11) |
| Intra-abdominal | 22(17) | 4(14) | 15(17) | 56(27) | 116(31) | 98(18) |  | | | 40(20) | 33(22) | 70(14) | 29(22) | 85(25) | 84(33) | 2(4) | 13(23) | 4(9) |
| Pneumonia |  |  |  | 2(14) | 42(11) | 79(14) |  | | | 22(11) | 28(19) | 55(12) | 27(21) | 87(25) | 39(15) | 5(10) | 18(32) | 5(11) |
| Soft tissue |  |  |  | 6(3) | 13(3) | 15(3) |  | | | 10(5) | 8(5) | 21(4) |  |  |  |  |  |  |
| Endocarditis |  |  |  |  |  |  |  | | | 9(5) | 8(5) | 6(1) |  |  |  | 0(0) | 1(2) | 0(0) |
| Other |  |  |  | 3(1) | 68(18) | 37(7) |  | | | 4(2) | 5(3) | 8(2) | 21(16) | 71(21) | 28(11) |  |  |  |
| Unknown | 27(21) | 6(21) | 32(36) |  |  |  |  | | | 45(23) | 22(15) | 127(27) | 21(16) | 59(17) | 48 (19) |  |  |  |
| Risk of bias | High | | | Low | | | High | | | Moderate | | | High | | | Low | | |

*p value refers to comparison of HCAI with CAI or HCAI with HAI

# PDR = MRSA, *Pseudomonas* sp, *Acinectobacter* sp, *Stenotrophomonas maltophilia*, ESBL

**1.** Umeki K, Tokimatsu I, Yasuda C, et al. Clinical features of healthcare-associated pneumonia (HCAP) in a Japanese community hospital: Comparisons among nursing home-acquired pneumonia (NHAP), HCAP other than NHAP, and community-acquired pneumonia. *Respirology.* Jul 2011;16(5):856-861.

**2.** Park SC, Kang YA, Park BH, et al. Poor prediction of potentially drug-resistant pathogens using current criteria of health care-associated pneumonia. *Respiratory Medicine.* Sep 2012;106(9):1311-1319.

**3.** Shindo Y, Sato S, Maruyama E, et al. Health-Care-Associated Pneumonia Among Hospitalized Patients in a Japanese Community Hospital. *Chest.* Mar 2009;135(3):633-640.

**4.** Seki M, Hashiguchi K, Tanaka A, et al. Characteristics and disease severity of healthcare-associated pneumonia among patients in a hospital in Kitakyushu, Japan. *Journal of Infection and Chemotherapy.* Jun 2011;17(3):363-369.

**5.** Garcia-Vidal C, Viasus D, Roset A, et al. Low incidence of multidrug-resistant organisms in patients with healthcare-associated pneumonia requiring hospitalization. *Clinical Microbiology and Infection.* 2011;17(11):1659-1665.

**6.** Jung JY, Park MS, Kim YS, et al. Healthcare-associated pneumonia among hospitalized patients in a Korean tertiary hospital. *Bmc Infectious Diseases.* Mar 11 2011;11.

**7.** Jeon EJ, Cho S-G, Shin JW, et al. The Difference in Clinical Presentations between Healthcare-Associated and Community-Acquired Pneumonia in University-Affiliated Hospital in Korea. *Yonsei Medical Journal.* Mar 1 2011;52(2):282-287.

**8.** Cardoso T, Ribeiro O, Aragao I, Costa-Pereira A, Sarmento A. Differences in microbiological profile between community-acquired, healthcare-associated and hospital-acquired infections. *Acta Med Port.* Jul-Aug 2013;26(4):377-384.

**9.** Lee JH, Kim YH. Comparison of clinical characteristics between healthcare-associated pneumonia and community-acquired pneumonia in patients admitted to secondary hospitals. *Brazilian Journal of Infectious Diseases.* Jul-Aug 2012;16(4):321-328.

**10.** Depuydt P, Putman B, Benoit D, Buylaert W, De Paepe P. Nursing home residence is the main risk factor for increased mortality in healthcare-associated pneumonia. *J Hosp Infect.* Feb 2011;77(2):138-142.

**11.** Pascual V, Salvado M, Calbo E, et al. Healthcare-Associated Pneumonia: A Category Under Review. *Abstracts of the Interscience Conference on Antimicrobial Agents and Chemotherapy.* 2010 2010;50.

**12.** Park HK, Song J-U, Um S-W, et al. Clinical characteristics of health care-associated pneumonia in a Korean teaching hospital. *Respiratory Medicine.* Nov 2010;104(11):1729-1735.

**13.** Carratalà J, Mykietiuk A, Fernández-Sabé N, et al. Health care-associated pneumonia requiring hospital admission: Epidemiology, antibiotic therapy, and clinical outcomes. *Archives of Internal Medicine.* 2007;167(13):1393-1399.

**14.** Wu K-S, Lee SS-J, Tsai H-C, et al. Non-nosocomial healthcare-associated infective endocarditis in Taiwan: an underrecognized disease with poor outcome. *Bmc Infectious Diseases.* Aug 17 2011;11.

**15.** Benito N, Miro JM, de Lazzari E, et al. Health Care-Associated Native Valve Endocarditis: Importance of Non-nosocomial Acquisition. *Annals of Internal Medicine.* May 5 2009;150(9):586-U585.

**16.** Aguilar-Duran S, Horcajada JP, Sorlí L, et al. Community-onset healthcare-related urinary tract infections: Comparison with community and hospital-acquired urinary tract infections. *Journal of Infection.* 2012;64(5):478-483.

**17.** Marschall J, Fraser VJ, Doherty J, Warren DK. Between Community and Hospital: Healthcare-Associated Gram-Negative Bacteremia among Hospitalized Patients. *Infection Control and Hospital Epidemiology.* Nov 2009;30(11):1050-1056.

**18.** Son JS, Song J-H, Ko KS, et al. Bloodstream Infections and Clinical Significance of Healthcare-associated Bacteremia: A Multicenter Surveillance Study in Korean Hospitals. *Journal of Korean Medical Science.* Jul 2010;25(7):992-998.

**19.** Evans CT, Hershow RC, Chin A, Foulis PR, Burns SP, Weaver FM. Bloodstream infections and setting of onset in persons with spinal cord injury and disorder. *Spinal Cord.* Aug 2009;47(8):610-615.

**20.** Rodriguez-Bano J, Lopez-Prieto MD, Portillo MM, et al. Epidemiology and clinical features of community-acquired, healthcare-associated and nosocomial bloodstream infections in tertiary-care and community hospitals. *Clinical Microbiology and Infection.* Sep 2010;16(9):1408-1413.

**21.** Vallés J, Alvarez-Lerma F, Palomar M, et al. Health-care-associated bloodstream infections at admission to the ICU. *Chest.* 2011;139(4):810-815.
