# Supplementary material for: Microbiology of healthcare-associated infections and the definition accuracy to predict infection by potentially drug resistant pathogens: a systematic review
Source: BMC Infect Dis. 2015 Dec 11;15:565. doi: 10.1186/s12879-015-1304-2 (PMC4676854; doi:10.1186/s12879-015-1304-2)
Supplement: Additional file 3: Table S2. — Studies with moderate or high risk of bias according to pre-defined criteria. (DOCX 36 kb) [file 12879_2015_1304_MOESM3_ESM.docx]

**Table S2 – Studies with moderate or high risk of bias according to pre-defined criteria**

| Study | Defines inclusion criteria | Defines selection method | Consecutive selection of patients | Attrition bias | Reporting bias | Overall |
| --- | --- | --- | --- | --- | --- | --- |
| Jung [^1^](#_ENREF_1) | yes | yes | yes | partial | partial | Moderate |
| Rodriguez-Bano [^2^](#_ENREF_2) | yes | yes | Not mentioned | partial | partial | Moderate |
| Park [^3^](#_ENREF_3) | yes | yes | yes | partial | partial | Moderate |
| Wu [^4^](#_ENREF_4) | yes | yes | yes | partial | partial | Moderate |
| Pascual [^5^](#_ENREF_5) | yes | yes | yes | yes | yes | High |
| Depuydt [^6^](#_ENREF_6) | yes | yes | yes | yes | yes | High |
| Seki [^7^](#_ENREF_7) | yes | yes | yes | yes | yes | High |
| Umeki [^8^](#_ENREF_8) | yes | yes | yes | yes | yes | High |
| Aguilar-Duran [^9^](#_ENREF_9) | yes | yes | yes | yes | yes | High |
| Evans [^10^](#_ENREF_10) | yes | yes | Not mentioned | yes | yes | High |
| Vallés [^11^](#_ENREF_11) | yes | yes | yes | yes | yes | High |
| Marschall [^12^](#_ENREF_12) | yes | yes | yes | yes | yes | High |

**1. Jung Y, Lee MJ, Shin HY, et al. Differences in characteristics between healthcareassociatedand community-acquired infection incommunity-onset Klebsiella pneumoniaebloodstream infection in Korea. *Bmc Infectious Diseases* 2012: 239.**

**2. Rodriguez-Bano J, Lopez-Prieto MD, Portillo MM, et al. Epidemiology and clinical features of community-acquired, healthcare-associated and nosocomial bloodstream infections in tertiary-care and community hospitals. *Clinical Microbiology and Infection* 2010; 16(9): 1408-13.**

**3. Park HK, Song J-U, Um S-W, et al. Clinical characteristics of health care-associated pneumonia in a Korean teaching hospital. *Respiratory Medicine* 2010; 104(11): 1729-35.**

**4. Wu K-S, Lee SS-J, Tsai H-C, et al. Non-nosocomial healthcare-associated infective endocarditis in Taiwan: an underrecognized disease with poor outcome. *Bmc Infectious Diseases* 2011; 11.**

**5. Pascual V, Salvado M, Calbo E, et al. Healthcare-Associated Pneumonia: A Category Under Review. *Abstracts of the Interscience Conference on Antimicrobial Agents and Chemotherapy* 2010; 50.**

**6. Angus DC, Linde-Zwirble WT, Lidicker J, Clermont G, Carcillo J, Pinsky MR. Epidemiology of severe sepsis in the United States: analysis of incidence, outcome, and associated costs of care. *Crit Care Med* 2001; 29(7): 1303-10.**

**7. Seki M, Hashiguchi K, Tanaka A, et al. Characteristics and disease severity of healthcare-associated pneumonia among patients in a hospital in Kitakyushu, Japan. *Journal of Infection and Chemotherapy* 2011; 17(3): 363-9.**

**8. Umeki K, Tokimatsu I, Yasuda C, et al. Clinical features of healthcare-associated pneumonia (HCAP) in a Japanese community hospital: Comparisons among nursing home-acquired pneumonia (NHAP), HCAP other than NHAP, and community-acquired pneumonia. *Respirology* 2011; 16(5): 856-61.**

**9. Aguilar-Duran S, Horcajada JP, Sorlí L, et al. Community-onset healthcare-related urinary tract infections: Comparison with community and hospital-acquired urinary tract infections. *Journal of Infection* 2012; 64(5): 478-83.**

**10. Evans CT, Hershow RC, Chin A, Foulis PR, Burns SP, Weaver FM. Bloodstream infections and setting of onset in persons with spinal cord injury and disorder. *Spinal Cord* 2009; 47(8): 610-5.**

**11. Vallés J, Alvarez-Lerma F, Palomar M, et al. Health-care-associated bloodstream infections at admission to the ICU. *Chest* 2011; 139(4): 810-5.**

**12. Marschall J, Fraser VJ, Doherty J, Warren DK. Between Community and Hospital: Healthcare-Associated Gram-Negative Bacteremia among Hospitalized Patients. *Infection Control and Hospital Epidemiology* 2009; 30(11): 1050-6.**
